# Surgical Treatment of Long-Standing Overt Ventriculomegaly in Adults (LOVA): A Comparative Case Series between Ventriculoperitoneal Shunt (VPS) and Endoscopic Third Ventriculostomy (ETV)

**DOI:** 10.3390/ijerph19041926

**Published:** 2022-02-09

**Authors:** Nicola Montemurro, Antonino Indaimo, Davide Tiziano Di Carlo, Nicola Benedetto, Paolo Perrini

**Affiliations:** 1 Department of Neurosurgery, Azienda Ospedaliera Universitaria Pisana (AOUP), 56100 Pisa, Italy; davide.dcr0@gmail.com (D.T.D.C.); nbenedetto@ao-pisa.toscana.it (N.B.); paolo.perrini@unipi.it (P.P.); 2 Department of Translational Research and New Technologies in Medicine and Surgery, University of Pisa, 56100 Pisa, Italy; 3 Department of Translational Medicine, University of Ferrara, 44121 Ferrara, Italy; indaimo.ant@gmail.com

**Keywords:** long-standing overt ventriculomegaly, LOVA, ventriculoperitoneal shunt, endoscopic third ventriculostomy, clinical outcome, traumatic brain injury

## Abstract

*Background:* Long-standing overt ventriculomegaly in adults (LOVA) is an uncommon type of adult chronic hydrocephalus. In recent years, conflicting case series described different outcomes after treatment of LOVA with endoscopic third ventriculostomy (ETV) or ventriculoperitoneal shunt (VPS). The aim of this study is to report a single institutional surgical experience of patients with LOVA in order to evaluate the clinical outcome of those patients treated with one or, sometimes, both surgical procedures, analyzing the main clinical features of these patients, before and after surgery. *Methods:* We conducted a retrospective study on 31 patients with diagnosis of LOVA, who were treated in our University Hospital between December 2010 and October 2020. We reported gender, age, clinical presentation, surgical treatment, and clinical outcome according to the Kiefer index (KI). Evans’ index, head circumference, aqueductal stenosis and expanded/destroyed sella turcica were assessed on preoperative MRI. *Results:* The most common clinical manifestation was gait disturbances (100%) followed by urinary incontinence in 23 (74.2%) patients and cognitive deficits in 22 (71%) patients. On preoperative MRI, the overall mean Evans’s Index was 0.49, whereas the overall mean head circumference was 57.3 cm. Twenty-three patients (74.2%) had obliterated cortical sulci, 20 (64.5%) patients had aqueductal stenosis, and 22 (71%) patients had an expanded/destroyed sella turcica on preoperative MRI. Fifteen (48.4%) patients underwent ETV and sixteen (51.6%) were treated with VPS as first surgical procedure. Four (26.6%) out of fifteen patients treated with ETV required a subsequent VPS. The overall median age of patients was 64 (IQR: 54.5–74) and the overall median follow-up was 57 months (IQR 21.5–81.5). Overall morbidity was 22.5%. Mean recovery index (RI), according to KI, was 3.8 ± 4.3 and 2.2 ± 5.6 (*p* = 0.05) at last follow-up in patients treated with ETV and VPS, respectively. *Conclusions:* The choice of surgical treatment of LOVA remains under discussion. Although EVT is a tempting option for patients with LOVA, conversion to VP shunt is not uncommon.

## 1. Introduction

Long-standing overt ventriculomegaly in adults (LOVA), as defined by Oi and colleagues [1] in 2000, is a type of hydrocephalus, which manifests in adults after a long and slow clinical course with symptoms of chronic hydrocephalus, a head circumference of more than two standard deviations above the 98th percentile, and overt triventriculomegaly on neuroimaging, in the absence of a secondary cause for aqueductal stenosis in adulthood. The mechanism of this phenomenon remains unclear, but it has been hypothesized that there is a partial obstruction of cerebrospinal fluid (CSF) flow through the aqueduct of Sylvius before fusion of cranial sutures followed by restoration of CSF flow before the occurrence of clinical symptoms [2,3]. This theory explains the large head circumference and initial asymptomatic course of patients with LOVA, until the hydrocephalus becomes decompensated and symptoms manifest in adults [1,4]. Diagnostic criteria include overt ventriculomegaly involving the lateral and III ventricle with obliterated cortical sulci and/or expanded or destroyed sella turcica detected on magnetic resonance imaging (MRI). The most common reported symptoms are macrocephaly, headache, sub-normal IQ, dementia, gait disturbance, urinary incontinence, vegetative state, akinetic mutism, apathetic consciousness, and parkinsonism [1].

In recent years, several papers reported conflicting results in patients with LOVA treated with endoscopic third ventriculostomy (ETV) or ventriculoperitoneal shunt (VPS) [2,5,6,7,8,9]. As each series advocate different opinions as to the optimal management for this chronic condition, the aim of this study is to report a single institutional experience to evaluate the clinical outcome after VP shunt or ETV in patients with LOVA.

## 2. Materials and Methods

### 2.1. Patient Population

We conducted a retrospective study of patients with a diagnosis of LOVA, who were treated in our University Hospital between December 2010 and October 2020. A head circumference of more than two standard deviations above the 98th percentile and overt triventriculomegaly on neuroimaging, in the absence of a secondary cause for aqueductal stenosis in adulthood, were required for diagnosis of LOVA. As is typical for retrospective studies, the choice of surgical technique (ETV vs. VPS) was at the discretion of the treating surgeon (with 8 to 22 years of surgical experience) and based on the literature of those years. We reported gender, age, clinical presentation, type of surgical procedure, and follow-up. Depression and schizophrenia were defined as the presence of five or more symptoms and two or more symptoms, respectively, according to the diagnostic criteria of the Diagnostic and Statistical Manual of Mental Disorders, Fourth Edition (DSM-IV) [10]. Similarly, bipolar disorders, anxiety, and disinhibition were defined according to the diagnostic criteria of the Diagnostic and Statistical Manual of Mental Disorders, Fourth Edition (DSM-IV) [11]. The Repeatable Battery for the Assessment of Neuropsychological Status (RBANS), according with Randolph et al. [12], was used to identify neuropsychological symptoms. RBANS combines five functional domains: recent memory, visual spatial and constructional ability, understanding of language, attention, and delayed memory. Parkinsonism was defined, according to Oi et al. [1], as the presence of tremor and akinesia or masked face.

These specific symptoms were assessed individually before surgery at time of diagnosis, then evaluated after surgery and at last follow-up, and they were grouped into four categories: normal-pressure hydrocephalus (NPH-like) symptoms (which include gait disturbance, urinary incontinence, and cognitive deficit), intracranial hypertension (IH)-like symptoms (which include headache and cognitive deficit), neuropsychological or psychiatric disorders, and parkinsonism, as described above [1,5]. We also evaluated the clinical outcome of these patients, immediately after surgery and at the last follow-up, using the Kiefer Index (KI) scale (Table 1) [3] and the recovery index (RI) according to the math equation below as described by Kiefer et al. [3]:

RI = (preoperative KI − postoperative KI) × 10/preoperative KI.

### 2.2. Image Analysis

Evans’ index, head circumference, the presence of obliterated cortical sulci, the aqueductal stenosis, and an expanded/destroyed sella turcica were assessed on preoperative MRI. In addition, two neurosurgeons independently assessed the presence of a disproportionately enlarged subarachnoid space hydrocephalus (DESH) [10], the callosal angle, and an enlarged cisterna magna measuring 10 mm or more on midsagittal images on preoperative MRI [13]. The images of each patient were analyzed using RadiAnt DICOM Viewer (version 2021.2, Medixant, Poznań, Poland), downloadable at https://www.radiantviewer.com, accessed on 25 January 2022), using the multiplanar reconstruction button. We used sets of DICOM images containing at least 64 slices.

### 2.3. Statistical Analysis

Statistical analysis was conducted to evaluate the impact of the patients’ clinical outcome in the postoperative period and at last follow-up, using descriptive statistics and t-test. Variables associated with outcome (*p* < 0.10) by univariate analysis were included in a multivariate Cox model using the step-wise method. Significance was fixed at 0.05 and all analyses were performed with SPSS version 26 (SPSS Inc. SPSS^®®^, Chicago, IL, USA).

## 3. Results

Between December 2010 and October 2020, 31 consecutive patients who fulfilled the criteria of LOVA underwent surgery at Neurosurgical Department of University Hospital of Pisa. Twenty-four were male (77.4%) and seven (22.6%) were female. The overall median age of patients at the time of diagnosis was 64 (IQR: 54.5–74). The median age of patients at the time of surgical treatment was 59 (IQR: 52.5–67) for ETV and 66.5 (IQR: 61–76) for VPS. Overall median follow-up was 57 months (IQR 21.5–81.5). All patients (100%) presented gait disturbance. Other clinical symptoms included urinary incontinence in 23 cases (74.2%), cognitive deficits in 22 patients (71%), memory deficit in 21 patients (67.7%), neuropsychological or psychiatric disorders in 17 cases (54.8%), dementia in 11 patients (35.5%), and headache and parkinsonism each in 10 patients (32.3%). After a preoperative MRI, the overall mean Evans’s Index was 0,49, whereas the overall mean head circumference was 57.3 cm. After a preoperative MRI, 23 patients (74.2%) had obliterated cortical sulci, 20 (64.5%) patients had aqueductal stenosis, 22 (71%) patients reported an expanded/destroyed sella turcica, 3 patients (9.7%) showed signs of DESH, and 7 patients (22.6%) had a wide cisterna magna. The preoperative mean callosal angle was 85.7°. Table 2 shows all the details.

**Figure 1:**
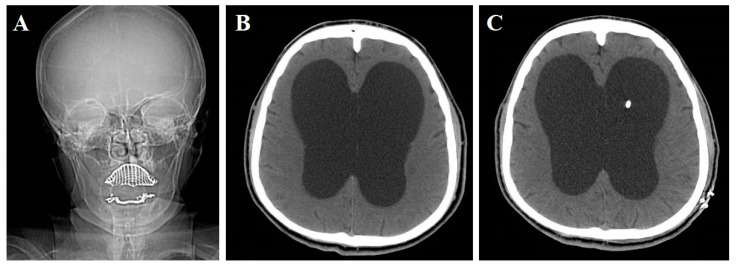
Preoperative skull X-ray showing slight macrocephaly (**A**), preoperative CT scan (**B**), and postoperative CT scan of a patient with LOVA that underwent VPS (**C**).

Fifteen (48.4%) patients underwent ETV and sixteen (51.6%) were treated with VPS (Figure 1). Four (26.6%) out of fifteen patients treated with ETV subsequently underwent rescue VPS because they did not improve or worsened at the last follow-up. The mean time between the first and second surgery was 17.5 months. In the group of patients treated with ETV, one patient (6.7%) developed a superficial wound infection. The overall morbidity of patients treated with VPS was 22.5%: one case presented wound infection, one case developed a chronic subdural hematoma (CSDH), and four cases presented shunt obstruction. In all four patients with shunt obstruction, a surgical revision was required. The mean preoperative KI was 7.3 ± 1.9 and 8.9 ± 1.8 in patients treated with ETV and VPS, respectively. The mean postoperative KI was 4.1 ± 2.6 after surgery and 4.7 ± 3.4 at last follow-up in patients treated with ETV. Similarly, the mean postoperative KI was 6.3 ± 4.2 after surgery and 6.9 ± 5.2 at last follow-up in patients treated with VPS. These data resulted in a mean RI, according to KI [3], of 3.8 ± 4.3 and 2.2 ± 5.6 at last follow-up in patients treated with ETV and VPS, respectively. Table 3 shows all the details.

**Figure 2:**
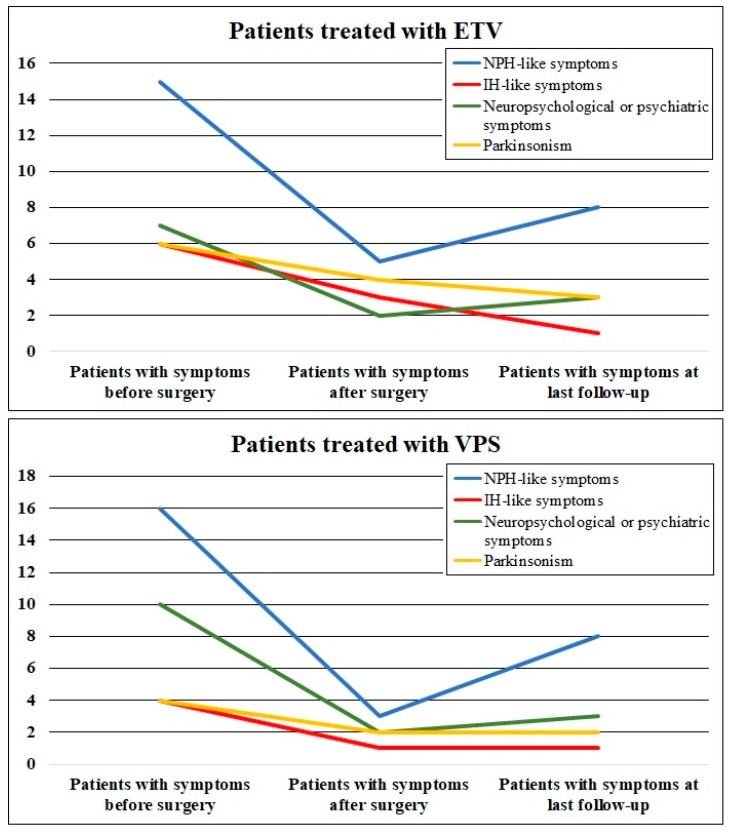
Shows the four symptom categories in patients treated with ETV (above) and VPS (below) before surgery, after surgery, and at last follow-up.

Preoperative symptoms of LOVA were divided into four categories: NPH-like symptoms, IH-like symptoms, neuropsychological or psychiatric symptoms, and parkinsonism. According to this classification, 15 (48.4%) out of 31 patients experienced improvement in NPH-like symptoms at last follow-up. Overall IH-like symptoms improved in 8 (80%) out of 10 patients, neuropsychological symptoms improved in 11 (64.7%) out of 17 patients, and parkinsonism improved in 5 (50%) out of 10 patients at last follow-up. Postoperative clinical improvement occurred in both groups (ETV and VPS), with no evidence of statistically significant differences (*p* = 0.05). Table 4 and Figure 2 show all the details.

## 4. Discussion

In 2000, Shizuo Oi et al. [1] originally defined the three diagnostic criteria for the diagnosis of LOVA: (1) overt ventriculomegaly involving the lateral and third ventricles with obliterated cortical sulci on CT/MR imaging; (2) clinical symptoms including macrocephaly, headaches, dementia, gait disturbance, urinary incontinence, vegetative state, akinetic mutism, apathetic consciousness, and parkinsonism; (3) neuroimages that demonstrate expanded or destroyed sella turcica as evidence of longstanding ventriculomegaly. Afterwards, Ibáñez-Botella et al. [6] set up the cut-off value of Evans Index > 0.4, indicating a real “overt ventriculomegaly” in patients with LOVA. 

The mysterious pathophysiology underlying LOVA makes Therapeutic decision-making for these patients complicated [2]. This condition is characterized by a progressive course and can lead to morbidity, with poor cognitive, psychological, and neurological outcomes [5]. However, despite this entity being described over 25 years ago, there is no consensus in the literature about the pathophysiology of LOVA [7]. The pathophysiology of LOVA seems to be traced back to aqueductal stenosis, with arrest of hydrocephalus before the onset of gross macrocephaly and the symptoms of raised ICP [2]. During the arrested hydrocephalic period, CSF flow is likely maintained via a combination of recanalization of the aqueduct, use of alternative CSF flow routes, and modification of CSF production or absorption [2,5]. This asymptomatic period is followed by a failure of the compensatory processes, leading to the symptomatic phase of LOVA, which is typically progressive. Although Oi et al. [1] described that a complete aqueductal stenosis was not a mandatory feature for LOVA diagnosis, other authors have discarded this hypothesis [2,14,15]. Indeed, a complete aqueductal stenosis seems to identify a separate subtype of adult hydrocephalus, described as late-onset idiopathic aqueductal stenosis (LIAS) [16], whose diagnosis became more easier with the introduction of MRI by allowing the clear visualization of the aqueductal canal [17]. The developmental mechanism of LIAS remains unclear, although the membranous ependymal-like tissue observed in the aqueduct suggested the preceding existence of an inflammatory process around this region [18]. Nevertheless, according to some observations by Rekate [14], aqueductal stenosis may be secondary to the third ventricle distortion, thus explaining the unsatisfactory outcome of ETV in his patients. Fukara and Luciano [16] reported the chronic onset of symptoms in 31 consecutive patients with diagnosis of LIAS. In these patients, the overall success rate after ETV was 80% and younger patients experienced headaches more often than the elderly; younger patients generally presented with NPH symptoms [16].

Reports of persistent symptoms in patients with LOVA after both ETV or VPS shunt procedures have led to a theory that, in some cases, the pathological process in adulthood leading to CSF volume imbalance may actually take place more distally than the aqueduct and is, therefore, attributable to a phenomenon of failure of CSF re-absorption. However, creating a pathway between the third ventricle and the basal cisterns can be effective, according to several authors [2,6,7,9], to bypass the hypothesized intracisternal obstruction in the posterior cranial fossa distal to the fourth ventricle, with the aim to restore a more physiological CSF flow between the ventricles, the cistern system, and, therefore, the subarachnoid spaces. 

The role of an “intracisternal” obstruction in communicating hydrocephalus with a bulging of the third ventricle floor has already been hypothesized by Kehler [19] in 2003. Palandri et al. [7] reported seven patients (38.9%) with a bulging third ventricle floor, as radiological signs are often found in aqueductal stenosis and obstructive hydrocephalus. In our study, aqueductal stenosis was found in 64.5% of patients. Similarly to Palandri et al. [7], who reported DESH sign on preoperative MRI in 11.1% of patients, we found DESH in 9.7% of patients. Otherwise, while we found a wide cisterna magna in only seven patients (22.6%), Palandri et al. [7] showed that all 18 patients (100%) included in their study showed an enlarged cisterna magna, suggesting the existence of an increased pressure in the cerebello-medullary cistern, pushing and shifting the cerebellar vermis upwards. It seems that this abnormality of CSF flow distally from the fourth ventricle and in the posterior cranial fossa with a concomitant patent aqueduct plays a pivotal role in the pathophysiology of the disease [5,7,20].

The clinical onset of LOVA can occur at any stage in adulthood, with a range between 22 and 81 years [2,5,21]. In our series, the overall median age of patients at the time of diagnosis was 64 (IQR: 54.5–74), whereas the median age of patients treated with ETV or with VPS at first was 59 (IQR: 52.5–67) and 66.5 (IQR: 61–76), respectively. These data are similar to previous studies [2,22,23,24] which reported a lower age in patients treated with ETV, probably due to the attitude to avoid a shunt dependency in younger patients. Al-Jumaily et al. [5] and Ved and al. [2] identified headaches and imbalance as the most common features in LOVA. Several studies [4,20,21,25] demonstrated that a majority of LOVA patients can achieve postoperative clinical improvement in headaches, dementia, motor skills, and neuropsychological or psychiatric disorders after ETV, as our study confirms. In our series, NPH-like preoperative symptoms were found in all patients. NPH-like symptoms were reported in only 51.6% of patients at last follow-up, with no statistically significant differences between the two groups of patients (*p* = 0.05).

As previous papers reported [6,20,21,25,26], we found that patients with LOVA may demonstrate a myriad of cognitive and psychological symptoms such as decline in memory, attention, and language skills, along with depression, anxiety, and disinhibition. All these symptoms may be difficult to identify clinically; nevertheless, when cognitive decline, inattention, and mood disturbances occur in LOVA, they can have significant impacts upon quality of life [2,5]. These symptoms were marked in 54.8% of patients of our series and both ETV and VPS led to an improvement of neuropsychological symptoms in half of the patients. Similar results were shown by Hamada et al. [20], who reported a significant improvement in the social functioning, memory, and mood after ETV. 

The parkinsonism usually occurred many months or years after the initial presentation of hydrocephalus [27]. Zeidler et al. [27] reported two cases of parkinsonism after recurrent obstructive hydrocephalus due to idiopathic aqueductal stenosis. Few previous papers [1,27] reported the rate of parkinsonisms, probably because this condition is usually incorporated within the more general frame of gait disturbance. In our series, we reported that parkinsonism improved in 5 (50%) out of 10 patients at last follow-up, suggesting that it was related to LOVA.

This study confirms that although surgical treatment of patients with LOVA can lead to clinical improvement of preoperative symptoms in more than half of patients, with our current knowledge, it is difficult to define which surgical option (ETV or VPS) can be considered the standard treatment of these patients. Ibáñez-Botella et al. [6], based on their experience on 27 adult patients with LOVA treated with ETV with a success rate of 76%, proposed ETV as the treatment of choice for patients with symptomatic LOVA, associated with Monro foraminoplasty (two cases) or septostomy (one case), when necessary. However, due to transitory success and failure in four patients (15%) and in two patients (7%) who underwent ETV, respectively, Ibáñez-Botella et al. [6] proposed a therapeutic algorithm including ETV in the case of aqueductal stenosis and VPS when a patent aqueduct was demonstrated. In the evaluation of success rate of ETV in LOVA, Oi successfully defined the restoration of flow through the stoma together with stable hydrocephalus (no clinical progression and stable or diminished ventricular size) [1,28]. On the other hand, Jenkinson et al. [8] successfully defined the presence of a clinical criterion (resolution of symptoms) together with a radiological criterion (presence of flow through the stoma). Rekate et al. [14] proposed that aqueductal stenosis can be a secondary phenomenon explaining the poor results of ETV in his series. Isaacs et al. [25] reported that ETV success rate as primary treatment modality for adult hydrocephalus of approximately 87% and a symptomatic improvement in 99% of patients after two ETVs.

As this study confirmed, the impact of surgery on clinical outcome of patients with LOVA is significantly relevant in the immediate postoperative period, while it tends to slightly decrease at last follow-up [5,6]. This most commonly occurs for NPH-like symptoms, whereas IH-symptoms, neuro-psychological symptoms, and parkinsonism usually maintain clinical improvement longer. Although VPS appears to improve NPH-like symptoms earlier in the first postoperative period compared to ETV, this difference in clinical outcome is lost at last follow-up. In accordance with the literature [1,2,6,8,14,22,24,25], although ETV seems to be preferred in term of lower surgical complications compared to VPS with similar clinical outcome, ETV required a conversion to VPS and a second surgery in 26.7% of our patients. Similarly, Al-Jumaily et al. [5] reported that ETV was successful in controlling symptoms in 89% of patients with three patients (15%) requiring shunts (gravitational valves). Gravitational shunts, as opposed to conventional DP shunt, can effectively prevent over-drainage even in this high-risk group with LOVA hydrocephalus [29]. Kiefer et al. [29] reported in their series that all 30 patients with LOVA received gravitational shunt valves. Currently, there are two technical types of gravitational shunt valves available, of which the first is the “switcher” type, in which there are two valves in one housing (a low-pressure valve for the prone position and a high-pressure valve for the erect position), and the second one is the hydrostatic or ‘‘counter balancer” type, in which the weight of the hanging CSF column (hydrostatic pressure) is counter balanced by the weight of a tantalum ball, which closes a cone dependent on the body’s angle [29]. As there is no scientific evidence for the clinical effects of these two theoretical valve mechanical function considerations, Kiefer et al. [29] suggested to use the more expensive “counter balancer” type in patients with a very large discrepancy between brain and skull volume (high-risk patients with thin residual cortex) and, otherwise, to use the cheaper “switcher” type in patients deemed to be at lower risk of over-drainage. Future studies are needed to better understand the pathophysiology of LOVA.

### Limitations of the Study

The current study is a retrospective analysis of a nonrandomized consecutive case series of patients undergoing VPS or ETV for LOVA, according to the discretion of the treating surgeon. Although baseline demographics did not statistically differ between the groups, treatment allocation may affect the direct comparability between VPS or ETV groups. Finally, our study reflects a single-center experience, with patients treated at the same University Hospital, and was limited to the last 10 years.

## 5. Conclusions

Clinical presentation of LOVA includes NPH-like symptoms, IH-like symptoms, neuropsychological or psychiatric symptoms, and parkinsonism. The surgical treatment of patients with LOVA allows clinical improvement in most of the patients, but NPH-like symptoms can recur in more than 50% of patients. ETV and VPS offer similar clinical outcomes in these patients. ETV was associated with lower surgical complications and morbidity, although roughly 26% of patients required VPS conversion.

## Figures and Tables

**Table 1:** Kiefer index (KI) scale clinical presentation and outcome according to Kiefer et al. [3].

	Mental	Gait Disturbance	Incontinence	Headache	Dizziness
0	No apparent deficit	Not or only in special tests handicapped	None	None	None
1	Forgetful, impaired concentration	-	Urge-Incontinence	Intermittent (independent of severity) or moderate permanent headache	Intermittent spontaneous dizziness
2	-	Wide legged but per se safe gait	-	-	Permanent dizziness
3	-	-	Intermittent bladder incontinence	-	-
4	Apathy or only partially oriented	Troublesome gait, only with crutches possible	Permanent bladder incontinence	Permanent severe headache	
5	-	Only a view steps with the aid of one person possible	-	-	-
6	Totally disoriented	Unable to go	Bladder and bowel incontinence	-	-

**Table 2:** Demographic, clinical, and radiological data.

	ETV (%)	VPS (%)	Total (%)
Patients	15 (48.4)	16 (51.6)	31
Male	10 (41.7)	14 (58.3)	24
Female	5 (71.4)	2 (28.6)	7
Age at diagnosis (median, years)	59 (IQR: 52.5–67)	66.5 (IQR: 61–76)	64 (IQR: 54.5–74)
Follow-up (median, months)	49 (IQR: 19.5–69)	72.5 (IQR: 25–99.8)	57 (IQR: 21.5–81.5)
Clinical Presentation			
Headache	6 (40)	4 (25)	10 (32.3)
Dementia	5 (33.3)	6 (37.5)	11 (35.5)
Cognitive Deficits	9 (60)	13 (81.3)	22 (71.0)
Gait Disturbance	15 (100)	16 (100)	31 (100)
Urinary Incontinence	10 (66.7)	13 (81.3)	23 (74.2)
Memory deficits	9 (60)	12 (75)	21 (67.7)
Neuropsychological or psychiatric disorders	7 (46.7)	10 (62.5)	17 (54.8)
Parkinsonism	6 (40)	4 (25)	10 (32.3)
Radiological Features			
Evans Index (mean)	0,5	0,49	0,49
Head circumference (mean, cm)	57,5	57,13	57,3
Obliterated cortical sulci	12 (80)	11 (68.8)	23 (74.2)
Aqueductal stenosis	11 (73.3)	9 (56.3)	20 (64.5)
Expanded/destroyed sella turcica	11 (73.3)	11 (68.8)	22 (71)
DESH	2 (13.3)	1 (6.25)	3 (9.7)
Callosal angle (mean, °)	84.8	86.6	85.7
Wide cisterna magna	4 (26.6%)	3 (18.8)	7 (22.6)

ETV, endoscopic third ventriculostomy; KI, Kiefer index; SD, standard deviation; n/a, not applicable; RI, recovery index; VPS, ventriculoperitoneal shunt.

**Table 3:** Postoperative complications and clinical outcome.

	ETV	VPS	Total	*p* Value
Surgical complications				
Postoperative infection (%)	1 (6.7)	1 (6.3)	2 (6.5)	-
CSDH (%)	0	1 (6.3)	1 (3.2)	-
Shunt Obstruction (%)	n/a	4 (25)	4 (12.9)	-
Surgical revision (%)	5 (33.3)	5 (31.3)	10 (32.3)	-
Conversion to VPS (%)	4 (26.7)	n/a	n/a	
Time between ETV and VPS (median, months)	17.5	n/a	n/a	-
Clinical Outcome				
Pre-operative KI (mean ± SD)	7.3 ± 1.9	8.9 ± 1.8	8.1 ± 2	*p* = 0.02
Post-operative KI (mean ± SD)	4.1 ± 2.6	6.3 ± 4.2	5.2 ± 3.6	*p* = 0.10
Post-operative KI at last FU (mean ± SD)	4.7 ± 3.4	6.9 ± 5.2	5.9 ± 4.5	*p* = 0.18
Post-operative RI of KI (mean ± SD)	4.07 ± 4.3	3.1 ± 4	3.6 ± 4.1	*p* = 0.50
RI of KI at last FU (mean ± SD)	3.8 ± 4.3	2.2 ± 5.6	3 ± 5	*p* = 0.58

CSDH, chronic subdural hematoma; ETV, endoscopic third ventriculostomy; FU, follow-up; KI, Kiefer index; RI, recovery index; SD, standard deviation; VPS, ventriculoperitoneal shunt.

**Table 4:** Clinical outcome with symptom evaluation.

	Surgical Procedure	Clinical Presentation before Surgery (%)	Clinical Outcome after Surgery (%)	Clinical Outcome at Last Follow-Up (%)	Pre-op KI	Post-op KI	Post-op KI at Last FU
NPH-like symptoms	ETV	15 (100%)	5 (33.3%)	8 (53.3%)	7.3	4.1	4.7
	VPS	16 (100%)	3 (18.8%)	8 (50%)	8.9	6.3	6.9
	TOT	31 (100%)	8 (25.8%)	16 (51.6%)	8.1	5.2	5.9
IH-like symptoms	ETV	6 (40%)	3 (20%)	1 (6.7%)	7.3	4.1	4.7
	VPS	4 (25%)	1 (6.3%)	1 (6.3%)	8.9	6.3	6.9
	TOT	10 (32.3%)	4 (12.9%)	2 (6.5%)	8.1	5.2	5.9
Neuropsychological or psychiatric symptoms	ETV	7 (46.7%)	2 (13.3%)	3 (20%)	7.3	4.1	4.7
	VPS	10 (62.5%)	2 (12.5%)	3 (18.8%)	8.9	6.3	6.9
	TOT	17 (54.8%)	4 (12.9%)	6 (19.4%)	8.1	5.2	5.9
Parkinsonism	ETV	6 (40%)	4 (26.7%)	3 (20%)	7.3	4.1	4.7
	VPS	4 (25%)	2 (12.5%)	2 (12.5%)	8.9	6.3	6.9
	TOT	10 (32.3%)	6 (19.4%)	5 (16.1%)	8.1	5.2	5.9

ETV, endoscopic third ventriculostomy; IH, intracranial hypertension; VPS, ventriculoperitoneal shunt.
